# Supplementary material for: Are carbon emissions trading and green financial instruments synergistic? -Comprehensive quantitative research based on content analysis
Source: PLoS One. 2024 Mar 7;19(3):e0298601. doi: 10.1371/journal.pone.0298601 (PMC10919601; doi:10.1371/journal.pone.0298601)
Supplement: S1 Data — (ZIP) [file pone.0298601.s001.zip › Data/地级市Z_F工作报告.zip!/╜¡╦╒/│ú╓▌╩╨/└┤╫╘╣1⁄2╓┌║┼í╛┬φ┐╦╩2╛▌═°í┐_2016.docx]

各位代表：

现在，我代表常州市人民政府向大会作工作报告，请予审议，并请市政协各位委员和其他列席人员提出意见。

一、“十二五”时期和2015年工作回顾

“十二五”以来，市政府在中共常州市委的正确领导下，在市人大、市政协的监督支持下，紧紧依靠全市人民，主动适应经济发展新常态，解放思想，开拓创新，真抓实干，奋力拼搏，实现了经济社会持续稳定发展。我市获得全国文明城市、国家历史文化名城、国家生态市、中国人居环境奖等荣誉称号。

(一)综合实力明显提升。“十二五”期间全市地区生产总值先后迈上3000亿元、4000亿元、5000亿元台阶，年均增长10.7%，人均地区生产总值超过11万元。2015年一般公共预算收入比2010年增长62.8%，五年年均增长13%;社会消费品零售总额比2010年增长88.3%，五年年均增长13.5%。五年完成固定资产投资、外贸进出口总额、注册外资实际到账分别达1.5万亿元、1437.4亿美元、150亿美元，分别是“十一五”时期的2倍、1.8倍、1.5倍。

(二)转型升级成效显著。大力推进“三位一体”工业经济转型升级战略和现代服务业三年行动计划，提高经济发展的质量和效益，三次产业结构由2010年的3.3︰55.3︰41.4调整到2015年的2.8︰48.2︰49，重工业比重由2010年的78.8%调整到2015年的76.8%。预计 2015年规模以上工业总产值达11450亿元，是2010年的1.5倍;“十大产业链”产值占规模以上工业产值比重达33.3%。重大项目引进取得突破性进展，组织开展“重大项目突破年”、“重大项目深化年”活动，东风、众泰、北汽三个汽车整车项目和斯太尔发动机等一批汽车零部件项目落户我市，北京通航等通用航空项目成功落地。上市企业数量从2010年的21家增加到2015年的 38家，新三板挂牌企业达60家。预计2015年国内外旅游总收入达733亿元，是2010年的2.1倍。农业现代化水平指数跃居全省第二，被认定为国家现代农业示范区，水稻单产连续保持全省第一，成功举办第八届中国花卉博览会。

(三)发展活力日益增强。重要领域改革深入推进，按照“5416”基本框架推进行政审批制度改革，推进企业登记注册“三证合一、一照一码”和建设项目并联审批，市级行政审批事项由2011年的380项减少到2015年的213项，行政审批效率得到提高。产城融合综合改革积极推进，部分区域已形成基本形态。金融改革创新取得积极进展，成功组建运营江南农村商业银行、江南金融租赁公司，创业投资机构达89家。农村改革深入推进，土地承包经营权确权登记完成 70%，武进区全国农村改革试点工作有序推进。大力推进苏南国家自主创新示范区和创新型城市建设，实施“千名海外人才集聚工程”和“龙城英才计划”，企业创新地位更加突出，创新平台功能大幅提升，创新动力进一步增强。预计2015年全市研发经费支出占地区生产总值的比重达2.65%。2015年末，全市孵化器、加速器面积累计超过813万平方米，企业研发机构超过1240家，引进领军型创新创业人才超过1400名，每万名劳动者中高技能人才数居全省第一。连续14年荣获“全国科技进步先进市”称号，成为国家知识产权示范城市。武进高新区升格为国家级高新区。常州科教城创成国家级两化深度融合试验区，成为 “全国高职教育综合改革试验区”，并连续三年获评中国最佳创新园区第二名。实施产业、科技、项目、基础设施“四个西进”和区县挂钩合作，县域经济发展的质量和水平明显提升。

(四)城乡环境更加宜居。推进城市建设由“筑形态”向“优品质”转变，城市功能板块建设步伐加快，新龙国际商务城、钟楼新城、凤凰新城等城市新片区建设成效显著。京沪高铁常州北站、泰州大桥南接线、西绕城高速、青洋路高架北延、城市防洪包围圈、锡溧漕河等一批重大基础设施建成投运。建成新堂北路、永宁路、飞龙西路等一批城市道路。常州机场完成4E级改造并实现一类口岸开放。“9313”综合整治工程稳步推进，成为首批“江苏省优秀管理城市”。五年新增城乡绿地5000公顷以上，建成区绿化覆盖率达43%，生态绿城建设初见成效。村庄环境综合整治深入开展，建成一批美丽乡村示范村。大气环境整治成效明显，2015年全市空气质量优良率达70.7%，市区PM2 . 5平均浓度较2013年下降18.1%。全市饮用水源地水质达标率稳定保持100%。

(五)人民生活持续改善。预计2015年城乡居民人均可支配收入分别达42700元、22000元，比2010年分别增长66.5%、78.6%。五年累计新增就业60.1万人，援助困难群体就业5.2万人，城镇登记失业率控制在4%以内，成为全国创业先进城市。养老、医疗、失业三大保险综合覆盖率稳定在98%以上，企业退休人员养老金年均保持10%左右的幅度增长。在全省率先实现中心城区中低收入住房困难家庭应保尽保，成为省住房保障体系建设示范市。全市教育现代化建设监测得分位居全省第三位，江苏工业学院成功更名为常州大学。建成15分钟社区医疗卫生服务体系和省首批公共体育服务体系示范区，社区居家养老服务中心(站)实现全覆盖。中国大运河常州城区段成为世界遗产，文化场馆全部向社会免费开放，“文化100”、“文明社区天天乐”等活动让广大市民享受到高质量的文化服务。城市居民公交出行率达28%以上，连续保持“全国交通畅通工程”A类管理水平。法治建设加快推进，荣获全国社会治安综合治理最高奖“长安杯”。深入推进社会治理创新，城乡社区“减负增效”走在全省前列。妥善化解社会矛盾，社会大局保持和谐稳定。持续办好为民办实事项目，提高了人民群众的幸福感和满意度。

(六)政府建设全面加强。扎实开展政府系统党的群众路线教育实践活动和“三严三实”专题教育，严格执行中央八项规定和省、市有关精神，“四风”问题得到有效解决。深化政务公开，建立完善行政权力网上公开透明运行系统，市级部门预决算和市级“三公”经费预算全部公开。严格落实党风廉政建设责任制，持之以恒正风肃纪，坚定不移惩治腐败，政府系统反腐倡廉工作取得实效。着力推进依法行政，自觉接受市人大、政协及各民主党派的监督，五年来，共办理人大代表建议、政协提案2577件，妥善解决了一批经济社会发展方面的问题。

与此同时，外事侨务、对台事务、审计统计、人武人防、双拥优抚、海关国检、药监质监、国资国企、粮食物价、边防海事、档案方志、防灾减灾、安全生产、民族宗教、老龄、妇女儿童、残疾人、关心下一代等工作都取得了新成绩。

去年，面对错综复杂的国内外宏观环境和艰巨繁重的改革发展任务，我们不断创新工作举措，转变工作作风，狠抓工作落实，经济社会发展各项工作都取得了新成效。预计实现地区生产总值5300亿元，增长9%左右;一般公共预算收入466.3亿元，增长7.5%;固定资产投资3650亿元，增长10%左右;社会消费品零售总额达1980亿元，增长10%左右;注册外资实际到账24.87亿美元，比上年确认数略有增长;外贸出口1320亿元，同比增长0.6%。一是着力狠抓重大项目。引进了中航锂电、瑞声射频模组等3个投资超100亿元或10亿美元项目，以及北汽集团常州产业基地、中兴能源云计算华东基地、百度大数据产业园等6个超50亿元或5亿美元项目，北汽集团新能源汽车动力电池、艾莱奥特通用飞机整机制造等项目落户我市，众泰汽车、东风整车顺利下线，查特深冷LNG储罐一期、爱科农机等大项目竣工投运。二是加快经济转型升级步伐。十大产业链企业完成产值对全市规模以上工业经济增长的贡献率超过50%，碳材料、太阳能光伏、轨道交通、汽车及零部件等产业链快速发展。固定资产增值税抵扣超过30亿元，增幅列沿江八市之首。金融业增加值、电子商务交易额均实现两位数增长，12301国家智慧旅游公共服务平台基本建成，我市成为全国唯一的“旅游+互联网”创新示范城市，天目湖旅游度假区被评为首批国家级旅游度假区。苏南国家自主创新示范区建设全面推进，预计高新技术产业产值占规模以上工业比重达43.5%左右，高新技术企业达1126家，新增产学研合作项目1000项以上。省智能装备产业技术创新中心落户科教城。三是深入推进改革开放。圆满完成部分行政区划调整，生产力布局进一步优化。在全省率先公布市政府部门责任清单，完成市县两级政府机构改革任务，不动产统一登记制度改革全面实施。深化医药卫生体制改革，所有城市公立医院实施药品零差率销售。常州出口加工区、武进出口加工区获批综合保税区并通过验收。我市成为大陆游客赴台湾自由行城市，常州机场新开通日本、台湾等多条境外航线。西太湖科技产业园获批筹建省级高新区。四是提高城乡建设管理水平。常溧高速、延政西路西延、金武路快速化改造建成通车，地铁1号线等在建工程进展顺利，新孟河工程金坛先导段启动实施。推进生态绿城建设和国家森林城市创建，建成环高架、环湖东路等生态绿道，基本建成横塘河湿地公园、新龙生态林等多个公园绿地，实现扩绿4万亩，连网600公里，全市森林覆盖率提高0.8个百分点。大力开展城市环境综合整治，城市精细化管理水平得到提高。成功创成国家级节水型城市。科学应对、成功抗击百年不遇的洪涝灾害。五是加大社会事业建设力度。实施108个中小学布局调整和校安工程项目，就读四星级高中人数超过高中生总数的70%;高考本二以上达线率达77.46%，比上年提高6个百分点。青果巷历史文化街区保护利用工程顺利推进，恽代英纪念馆建成开放;文化产业增加值占地区生产总值的比重达5.63%，列全省第二。免费提供12类45项基本公共卫生服务，各辖市(区)全面创成全国基层中医药工作先进单位，城乡居民基本医疗保险管理体制实现整合。为民办实事各项任务全面完成。

各位代表!近五年发展取得的成就来之不易，这是全市上下在省委、省政府和市委的正确领导下，团结拼搏、攻坚克难、开拓创新的结果。在此，我代表市人民政府，向辛勤工作在各个领域的全市人民，向市人大代表、政协委员，向市各民主党派、工商联、各人民团体、各界人士和离退休老同志，向驻常人民解放军、武警官兵、公安政法干警和部省驻常单位，向所有关心、支持、参与常州建设和发展的中外投资者、科技工作者、务工人员和海内外朋友们，致以衷心的感谢和崇高的敬意!

在肯定成绩的同时，我们也清醒地看到工作中存在的问题和不足。主要表现在：经济结构依然偏重，调结构、转方式、增效益任务比较艰巨;重点领域和关键环节改革攻坚步伐有待加快，开放型经济发展水平需要进一步提升;基本公共服务与群众期望还有一定差距，生态环境保护力度有待加大;政府职能转变还要加快，作风建设、廉政建设、法治建设需要进一步增强。我们一定高度重视这些问题，采取更加有力的措施，切实加以解决。二、“十三五”时期的发展构想和2016年主要工作

从今年开始的“十三五”时期，是常州在全面建成小康社会的基础上，积极探索开启基本现代化建设新征程的重要阶段。我们将围绕“四个全面”战略布局，牢牢把握五大发展理念，坚持发展是第一要务，以提高发展质量和效益为中心，深入实施五大战略，努力打造全国一流的智能制造名城、长三角特色鲜明的产业技术创新中心和国内领先的产城融合示范区，基本建成具有突出竞争力和影响力的区域中心城市，加快建设经济强、百姓富、环境美、社会文明程度高的新常州。

“十三五”期间，全市经济社会发展总的要求是：

——在经济发展上，以重大项目为抓手，以创新驱动为主引擎，深入推进创新驱动发展，加快构建现代产业体系，拓展经济发展新空间，全面提升开放水平，厚植实体经济发展新优势，持续打造“常州智造创新版”和“常州服务升级版”，实现经济保持中高速增长、产业迈上中高端水平，提前实现地区生产总值比2010 年翻一番。

——在民生建设上，按照人人参与、人人尽力、人人享有的要求，坚守底线、突出重点、完善制度、引导预期，深入实施民生幸福工程，更实举措增加城乡居民收入，提前实现城乡居民收入比2010年翻一番，更大力度促进就业创业发展，更新起点提升公共服务能力，更高水平完善社会保障制度，扎实办好各项民生实事，提高幸福常州建设水平，使广大市民在共建共享发展中有更多获得感。

——在环境建设上，围绕打造自然环境之美、景观风貌之美、城乡协调之美，深入实施生态文明建设工程，有序完善空间形态，持续加强城乡建设，大力改善环境质量，加快构建生态文明制度体系，推动形成绿色发展方式和生活方式，建设美丽宜居城市。

——在社会文明程度建设上，顺应社会发展进步的需要，围绕建设文化强市，加强精神文明引领，建设江南文化名城，加强社会治理创新，推动物质文明和精神文明协调发展，不断提升市民文明素质和社会文明程度，努力构筑思想文化建设高地和道德风尚建设高地。

——在全面深化改革上，认真贯彻中央、省、市委关于改革的各项部署，坚持社会主义市场经济改革方向，充分发挥市场在资源配置中的决定性作用和更好发挥政府作用，科学把握改革的重点和主攻方向，积极推进改革试点试验，协调推进经济、政治、文化、社会、生态文明体制改革，不断激发体制机制新活力。

——在全面依法治市上，坚持依法治市、依法执政、依法行政共同推进，法治常州、法治政府、法治社会一起建设，加强政府立法工作，全面推动政府工作规范化、程序化、法治化建设，增强全社会特别是政府工作人员尊法学法守法用法观念，在全社会形成良好法治氛围和法治习惯，加快全市经济、政治、文化、社会、生态文明建设的法治化进程。

根据市委十一届十次全体会议的精神，市政府编制了《常州市国民经济和社会发展第十三个五年规划纲要》，现已印发给各位代表，提请大会审议。

今年是“十三五”发展的开局之年，也是践行五大发展理念、建设“强富美高”新常州的重要之年。今年政府工作的总体要求是：全面贯彻党的十八大、十八届三中、四中、五中全会精神，深入贯彻习近平总书记系列重要讲话精神，按照市委十一届十次全会部署，适应经济发展新常态，坚持改革开放，坚持稳中求进工作总基调，坚持稳增长、调结构、惠民生、防风险，落实宏观政策要稳、产业政策要准、微观政策要活、改革政策要实、社会政策要托底的要求，着力加强结构性改革，在适度扩大总需求的同时，去产能、去库存、去杠杆、降成本、补短板，扎实开展“重大项目推进年”活动，更大力度推进转型创新、产城融合、改革开放、生态文明、民生保障等各项工作，促进经济平稳健康发展和社会和谐稳定。

全市主要预期目标是：地区生产总值增长8.5%左右;一般公共预算收入增长6.5%左右;固定资产投资增长10%左右;社会消费品零售总额增长10%左右;外贸进出口总额和注册外资实际到账保持适度增长;城乡居民收入增长与经济增长同步;居民消费价格总水平涨幅控制在3%左右;城镇登记失业率控制在4%以内;全面完成省下达的节能减排目标任务。

为实现上述目标，我们将重点抓好八个方面工作：

(一)持续推进重大项目，着力强化发展支撑

1、加快建设速度。以更大力度持之以恒推进重大项目，加快重大项目建设进程，不断优化投资结构，为经济增长和转型升级提供强劲动力。积极实施156个省、市重点项目，确保续建项目竣工率60%，新建项目开工率100%。按照产业项目“六个一批”、社会事业和基础设施项目“四个一批”要求，推进重大项目分类管理，推动东风乘用车一期、同方威视等重大项目投产见效，加快中航锂电、百度大数据产业园、新能源汽车充电桩等项目建设进程。

2、创新招引方式。狠抓十大产业链和现代服务业招商，努力实现智能制造、通用航空、金融服务、健康服务、旅游休闲等领域重大项目招引的新突破。加强新技术、新产业、新业态项目招引，形成大数据、碳材料等新兴产业集聚发展态势。发挥产业投资基金在招商引资中的作用，大力吸引境内外投资基金来常投资、开展资本运作，推动重大项目建设。

3、优化服务举措。进一步创新投资管理方式，优化项目审批流程，不断完善投资环境。创新重点领域投融资机制，深入推动银政企合作，运用好债券融资等金融工具，加快建设中小微企业低息统贷平台，着力解决项目建设和企业发展融资难题。大力推进节约集约用地，盘活存量土地，提高项目单位面积投资强度和产出水平，为项目建设拓展空间。

(二)大力促进转型升级，提升经济发展质态

1、推进制造业中高端发展。深入推进“三位一体”工业经济转型升级战略，实施好“中国制造2025”常州行动纲要，不断增强工业经济核心竞争力。加快十大产业链发展，对产业链加强分类指导，细化推进举措，推动产业链向供应链上下游和价值链两端延伸，提升产业层次和附加值，重点是巩固提升太阳能光伏、轨道交通、智能数控和机器人等产业链实力，完善汽车及零部件产业链，拓展碳材料、通用航空等产业链发展空间，力争十大产业链产值占规模以上工业比重达35% 左右。更大力度加快传统产业改造升级，引导企业加快技术改造，重视质量品牌，推动兼并重组，盘活存量资产，不断提升传统产业市场占有率。加大“十百千”创新型企业培育力度，加快发展壮大20多家创新型领军企业，推动200多家科技型上市培育企业发展，新增高新技术企业100家以上。

2、全面发展现代服务业。围绕推进100个服务业重点项目、培育100家左右服务业重点企业、建设30家重点服务业集聚区，推动制造业服务化，加大服务业结构优化、规模扩张、业态创新力度，力争服务业增加值占地区生产总值比重提高1个百分点左右。按照建设“金融三中心”的要求，加快金融商务区建设步伐，重点集聚股权投资和产业并购基金等金融业态;做强地方法人金融机构，完成企业股改150家，力争5家企业在主板上市、60家企业在新三板挂牌。深入推进国家电子商务示范城市创建，开展跨境贸易电子商务试点，电子商务交易额增长30%以上。加快东方盐湖城、航天探索中心等旅游项目建设进程，提升城市旅游、乡村旅游、休闲旅游发展水平。发展壮大文化创意产业，加大龙头文化企业和示范园区培育力度，文化产业增加值占地区生产总值比重达6%左右。

3、加快发展现代农业。深入推进国家现代农业示范区建设，加快实施新型经营主体培育、现代农业产业园区建设、农业产业链延伸、农产品质量建设等工程，走产出高效、产品安全、资源节约、环境友好的农业现代化道路，确保农业现代化建设继续走在全省前列。推动落实“1185”农业布局规划，促进农业主导产业集聚集约发展。全年新建高标准农田4万亩，新发展高效设施农业(渔业)2万亩，新认定和提升10个现代农业产业园区，新发展市级农业产业化龙头企业10家左右、市级示范家庭农场40家、星级示范合作社40家。

4、积极发展互联网经济。大力推进“互联网+”行动计划，促进互联网、大数据、云计算等产业发展，催生更多的新产业、新技术、新业态、新模式。大力发展互联网平台经济，抓好国家智慧旅游公共服务平台、华贸通外贸综合服务平台等建设与运营，积极拓展大宗商品现货交易、物流专业服务以及各类细分服务平台，鼓励企业利用互联网平台拓展市场空间、提升竞争能力。推进科教城移动互联产业园、西太湖互联网产业园、中兴能源云计算华东基地等发展载体建设，尽快形成互联网经济集聚发展态势。

(三)深入实施创新驱动，建设区域创新中心

1、建设高水平创新园区。加快苏南国家自主创新示范区建设，围绕“一核两区多园”的布局，提升园区创新能级，努力使园区成为自主创新的战略高地和抢占高技术产业制高点的前沿阵地。大力推进常州国家高新区、武进国家高新区争先进位，集聚更多创新资源。发挥科教城“创新之核”作用，加快建设江苏省智能装备产业技术创新中心、江苏中科院智能科学技术应用研究院、省产研院专业所等平台，争创国家级科技服务示范区，着力在智能、设计、信息三大领域形成完整的产业链和创新链。大力建设中以、中德创新园，努力将园区打造成以科技创新、产业技术创新为驱动的国际合作典范。强化园区服务功能，建设常州科技街，鼓励园区普遍建立“一平台三中心”，不断完善和提升园区的产业和创新服务功能。

2、加快培育创新创业主体。强化企业创新主体地位和主导作用，推动创新要素向企业聚集，形成一批具有较强竞争力的创新型领军企业。大力建设新型企业研发机构，新增市级以上企业研发机构100家，推进“光伏科学与技术国家重点实验室”等企业重点实验室建设，鼓励以产业链为基础、按创新链组建的企业技术创新联盟。深入推进“大众创业、万众创新”，积极发展众创、众包、众扶、众筹空间，累计培育10000家各类初创科技企业。

3、完善创新生态体系。大力推进“龙城英才计划”和“技能龙城”建设行动计划，吸引更多高层次和高技能人才及团队来常发展。建立健全科技金融服务体系，加快发展股权投资、科技贷款和科技保险，促进拨、投、贷、保深度融合，创新支持科技型小微企业的科技金融模式。深入建设国家知识产权示范城市，加大知识产权保护和利用力度，鼓励企业形成更多的核心自主知识产权。

(四)全面深化改革开放，释放体制机制活力

1、推进重点领域改革。推进产城融合综合改革，重点在城镇化投融资创新、城乡公共资源配置等方面探索路径，加快形成符合时代要求、具有常州特色的产城融合改革新路。加强供给侧结构改革，进一步化解过剩产能，加快实体经济发展，提高供给体系质量与效益。深化国有企业改革，稳步推进经营性国有资产集中统一监管。继续深化农村改革，基本完成土地承包经营权确权登记颁证工作，开展深化农村集体产权制度改革试点，全面推进辖市(区)农村产权交易市场建设，有序推进武进区的全国农村宅基地制度改革试点。深化供销合作社综合改革，发挥综合服务优势。

2、推动开发园区提档升级。围绕提升开发区影响力和竞争力，推动开发区转型发展，着力提升投入产出效益。推动开发区产业集聚和特色发展，常州高新区重点发展高端装备、通用航空和生物医药产业，武进高新区重点发展机器人与智能装备、节能环保和电子信息产业，金坛经开区重点发展光伏新能源、整车制造产业，溧阳经开区重点发展输变电、储能装备产业，常州经开区重点发展轨道交通、智能电网、新型特种材料产业，天宁、钟楼等开发区也要加快推动主导特色产业集聚发展，提升开发区竞争力。大力推进常州、金坛、溧阳经开区申报国家级经济开发区，支持金坛金城工业园、常州航空产业园申报省级开发区，建设好中芬绿色科技产业园、海峡两岸健康产业合作示范区等园区平台。推进开发区管理模式创新，进一步理顺各类开发区与行政区管理职能关系，形成权责明确、分工合理、精简高效的管理体制和运行机制。

3、提升经济国际化水平。抢抓国家“一带一路”、长江经济带等重大开放机遇，大力实施全方位开放战略，进一步推进“五个国际化”建设，努力培育开放竞争新优势。加大高端制造业和现代服务业领域外资项目引进力度，着力提高外资质量和效益。推动外贸向“优进优出”转型，加强十大产业链出口品牌培育和出口基地建设，提升机电产品和高新技术产品进出口比重，加快形成以技术、品牌、服务、质量为核心的外贸竞争优势。引导工程机械、轨道交通等行业加快“走出去”，支持龙头企业开展跨国经营和并购重组，建立境外研发机构、生产基地和营销网络。大力发展跨境电商、国际服务外包等新型业态，扩大服务贸易规模。加快完善电子口岸平台功能，积极推动综合保税区建设发展，提升和扩大口岸开放综合效应。

(五)积极推进新型城镇化，提升城市品质

1、优化城市功能布局。开展新一轮城市总体规划修编前期工作。提升中心城区现代化水平，统筹推进城市改造更新，促进现代商贸与文化旅游融合发展。高品质建设新龙国际商务城、钟楼新城、凤凰新城等新板块，加快常州经开区现代化城市建设步伐。推动中心城区与金坛城区融合发展。围绕建设江苏西南门户城市和宁杭发展带副中心城市的定位，加快完善溧阳城市功能。

2、加大城市建设管理力度。加快现代综合交通体系建设，开工建设常宜、溧高、苏锡常南部通道高速公路和沿江城际铁路，加快推进233国道常州段、 122省道常州东段等国省干线公路建设，实施312国道快速化改造前期工程。进一步完善城市路网，完成白云路、雕庄路等工程，推进劳动西路、大仓路等道路建设，有序推进地铁一号线建设，启动建设地铁二号线。加快公用事业发展，继续实施自来水深度处理工程及天然气利用三期工程，完成生活垃圾转运站二期、江边污水厂进水管网扩建等工程。加快海绵城市建设，探索建设城市综合管廊。优化提升区域防洪除涝体系，加快实施新沟河、新孟河延伸拓浚工程，推进京杭运河常州经开区段三级航道整治。健全城市长效综合管理考核体系，深入开展城市环境综合整治，对城郊结合部、城中村、棚户区、老旧小区等城市环境薄弱地段开展专项整治，改善城市环境面貌，提高城市管理科学化、标准化、精细化水平。

3、加快城乡一体发展步伐。全面落实强农惠农富农政策，推动城市基础设施、公共服务和社会事业向农村倾斜，促进城乡基本公共服务均等化。加强中心镇和小城镇建设，推进省经济发达镇改革试点和市级中心镇扩权强镇，建设一批功能现代、特色鲜明的现代化小城镇。加大美丽乡村示范村建设力度，推进传统村落民居和历史文化名村保护，不断提高新农村建设水平。

(六)加强生态文明建设，促进绿色低碳发展

1、提高生态文明建设水平。深入推进国家生态文明示范市创建，将绿色发展理念融入经济社会生活各领域全过程。加强主体功能区建设，调整完善全市土地利用总体规划，逐步形成定位更清晰、布局更合理的国土空间开发格局。充分盘活利用存量土地，严格处置闲置土地，提升节地水平和产出效益。更大力度推进绿色清洁生产和循环经济发展，加快低碳经济示范区建设，构建绿色低碳循环发展的产业体系。

2、加快生态绿城建设。围绕创建国家森林城市和国家生态园林城市，开展大规模城乡绿化，提升城乡绿量和绿化养护水平，建设“处处皆绿、人人可享”的宜居宜业宜游城市。按照“增核、扩绿、连网”的要求，持续推进生态源保护与建设、生态廊道等生态绿城六大建设工程，建设横山、环太湖等郊野公园，加快皇粮浜公园、新龙湖湿地公园等城镇公园绿地建设步伐，实施老运河保护及环境整治，打造集人文、自然、旅游于一体的旅游资源。按照项目生态化原则，不断增强城市生态功能，营造更多绿色空间。

3、大力推进环境整治。实行最严格的环境保护制度，进一步提高全市环境质量。加大治水力度，加强太湖流域水环境和城市河道整治，推进工业、农业、生活等污染点源和面源治理，切实改善水环境质量。加大治气力度，严格控制大气污染物排放总量，推进主城区重污染企业关闭搬迁，控制煤炭消费总量，提高钢铁、水泥等企业污染治理水平，加强机动车尾气、扬尘、秸秆焚烧等污染治理，有效改善空气质量。加大治土力度，积极推进土壤污染防治，开展农田土壤环境调查，加强重点区域、重点企业地块土壤监测，对工业企业污染场地土壤实施修复。

(七)注重保障和改善民生，优化提升公共服务

1、持续提升民生质量。深入推进居民收入倍增计划，促进城乡居民收入持续增长。坚持以创业带动就业，新增城镇就业8.5万人，扶持创业6000人。推进全民参保计划，加快主城区社会保险一体化步伐，稳步推进机关事业单位工作人员养老保险制度改革，养老、医疗、失业三大保险综合覆盖率保持在98%以上。落实城乡低保标准自然增长机制，扩大社会救助覆盖面，完成本轮建档立卡低收入农户脱贫工作。加大棚户区改造力度，进一步改善居民住房条件，促进房地产业平稳健康发展，全年住房公积金政策性低息贷款额达60亿元。推行公共租赁住房共有产权制度试点工作，确保中心城区中低收入住房困难家庭应保尽保。加快完善养老服务体系，积极实施医养融合发展，推动健康养老服务业集聚区建设，改造提升城乡养老机构，标准化城乡居家养老服务中心(站)覆盖率分别达50%、 30%。

2、协调发展各项事业。加快教育现代化建设，推进教育资源布局调整和现代化学校建设，实施学前教育保障工程，启动和实施义务教育“新优质学校”创建工程，教育发展水平和质量保持全省前列。大力发展文化事业，加强大运河文化走廊建设和文化遗存保护，推进孟河古镇参加中国江南水乡古镇联合申遗，加快青果巷历史文化街区保护利用和市文化广场项目建设，深入推进省级公共文化服务体系示范区创建，进一步加大文化惠民力度，开展好“文化100”、“文明社区天天乐”、“文化点亮生活”等文化活动。加大“健康常州”建设力度，进一步优化卫生计生资源配置，推动公立医院改革，加快建立分级诊疗体系，强化社区卫生服务功能，做好全面二孩政策实施，提升卫生计生服务水平。深入推进公共体育服务示范区建设，启动实施公共体育设施建设提升工程，办好西太湖国际半程马拉松等国内外大型赛事。巩固和深化军地军政共建，广泛开展全民国防教育，进一步提高双拥共建水平。支持工会、共青团、妇联等人民团体工作，进一步加强外事侨务、对台事务、档案方志、民族宗教、防灾减灾、应急管理、气象、残疾人事业和关心下一代等工作。

3、加强和创新社会治理。加强精神文明建设，深入开展道德讲堂活动，完善全国文明城市建设长效机制，不断提升市民文明素质和城市文明程度。深入推进社区减负，建设和谐社区，增强社区服务功能，推动政府治理与社会调节、居民自治良性互动。完善社会矛盾源头预防和调处化解机制，加强改进信访和调解工作，有效预防和化解矛盾纠纷。落实重大决策社会稳定风险评估制度，强化社会治安防控体系建设。加强食品药品监管，健全食品药品追溯、监测和诚信体系。完善和落实安全生产责任和管理制度，加强安全生产基础能力建设和长效管理，加大监管执法力度，及时排查化解安全隐患，坚决遏制重特大安全生产事故发生。

与此同时，今年我们将办好以“三优三安两提升”(优质教育、优质医疗、优质养老、安心食品药品、安心饮用水、安心社会治安、提升生态绿城建设水平、提升大气环境质量)为重点的民生实事，提升民生幸福和社会和谐水平。

(八)突出提升行政效能，切实加强政府自身建设

1、优化政府服务。深化行政审批制度改革，持续推进简政放权、放管结合、优化服务，提高行政效能。创新财政投入方式，加强与金融资本、社会资本的合作，进一步发挥财政服务经济发展的作用。扩大政府购买公共服务，鼓励社会资本参与公益性事业投资运营，提高公共服务供给效率。完善政务服务体系，加快电子政务建设，切实发挥“一办四中心”作用。

2、推进依法行政。坚持法定职责必须为，法无授权不可为，落实政府部门权力清单和责任清单制度，把依法行政贯穿于政府工作的各个层面、各个环节。完善重大行政决策程序，建立重大决策终身责任追究制度及责任倒查机制，积极推行政府法律顾问制度，发挥政府立法的作用。强化行政权力监督，自觉接受人大法律监督、工作监督及政协民主监督，主动接受司法监督、社会监督和舆论监督，加强审计监督。严格规范行政执法行为，开展相对集中行政许可权和综合行政执法体制改革，进一步提高政府执行力和公信力。

3、加强作风建设。深入贯彻中央八项规定和省、市有关精神，持续加大“四风”整治力度，推进作风建设常态化、长效化。加强政风行风建设，提振政府工作人员干事创业的精气神，以“钉钉子”精神全力抓好各项工作落实，形成敢于负责、敢于担当、敢于创新的时代风尚。严格落实党风廉政建设责任制，加强政府系统廉政建设和反腐败工作，坚持有腐必反、有贪必肃、违规必究，始终保持惩治腐败的高压态势。深入贯彻厉行节约、反对浪费各项规定，严控“三公”经费支出，把更多财力用在急需的民生事业上，更好树立为民、务实、清廉的政府形象。

各位代表，新的目标激励人心，新的任务艰巨光荣，新的征程催人奋进，让我们紧密团结在以习近平同志为总书记的党中央周围，在省委、省政府和市委的坚强领导下，紧紧依靠全市人民，坚定信心、鼓足干劲、开拓创新、扎实工作，为顺利完成“十三五”规划确定的各项目标任务，为建设“强富美高”新常州而努力奋斗!
